# Supplementary material for: Investigating the influence of physiologically relevant hydrostatic pressure on CHO cell batch culture
Source: Sci Rep. 2021 Jan 8;11:162. doi: 10.1038/s41598-020-80576-8 (PMC7794228; doi:10.1038/s41598-020-80576-8)
Supplement: Supplementary file 1 — Supplementary Information 1. [file 41598_2020_80576_MOESM1_ESM.zip › S1/IgG/18Dec 4th round 5%CO2/C Dec14.pdf]

===

Acq. Operator	: SYSTEM	Seq. Line :	2
Sample Operator	: SYSTEM		
Acq. Instrument	: HPLC-0039	Location :	2
Injection Date	: 12/18/2018 11:50:03 AM	Inj :	1
		Inj Volume :	100.000 µl

Acq. Method : C:\Users\Public\Documents\ChemStation\1\Data\CHO_IgG 2018-12-18 11-44-16 \CHO_IGG_LC.M
Last changed : 12/18/2018 11:44:20 AM by SYSTEM
Analysis Method : C:\Users\Public\Documents\ChemStation\1\Data\CHO_IgG 2018-12-18 11-44-16 \CHO_IGG_LC.M (Sequence Method)
Last changed : 12/18/2018 11:48:43 AM by SYSTEM
(modified after loading) (Current integration events modified)

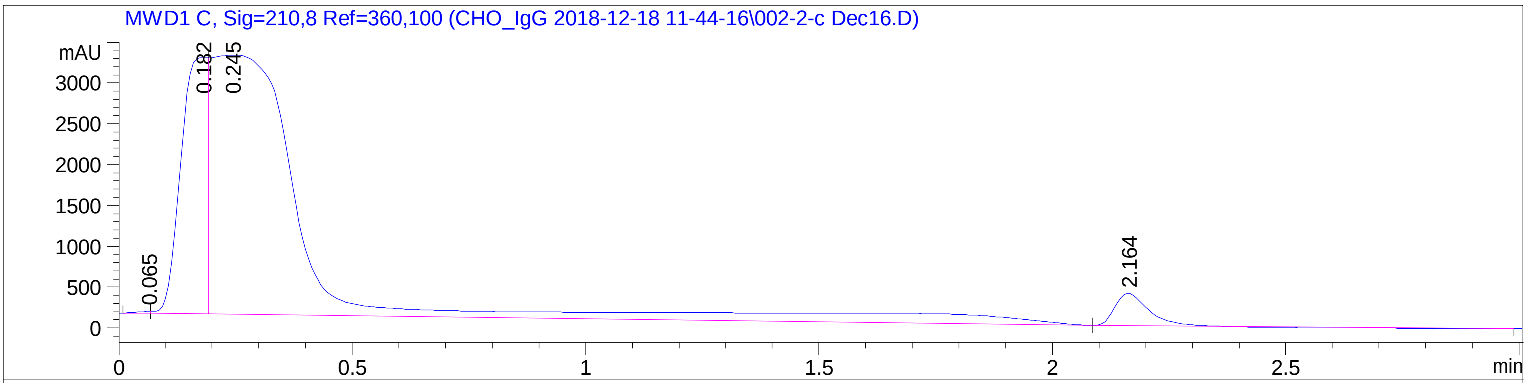

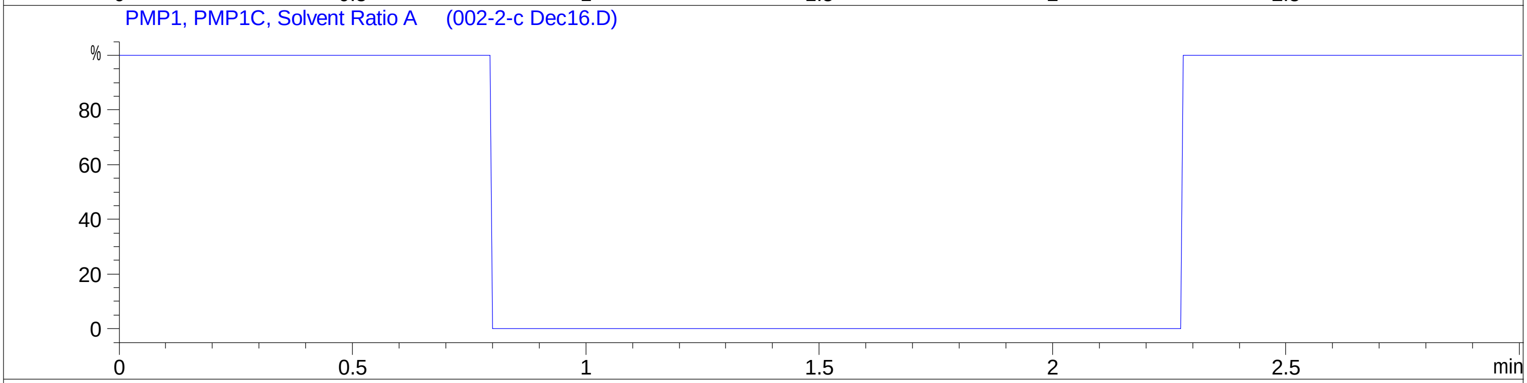

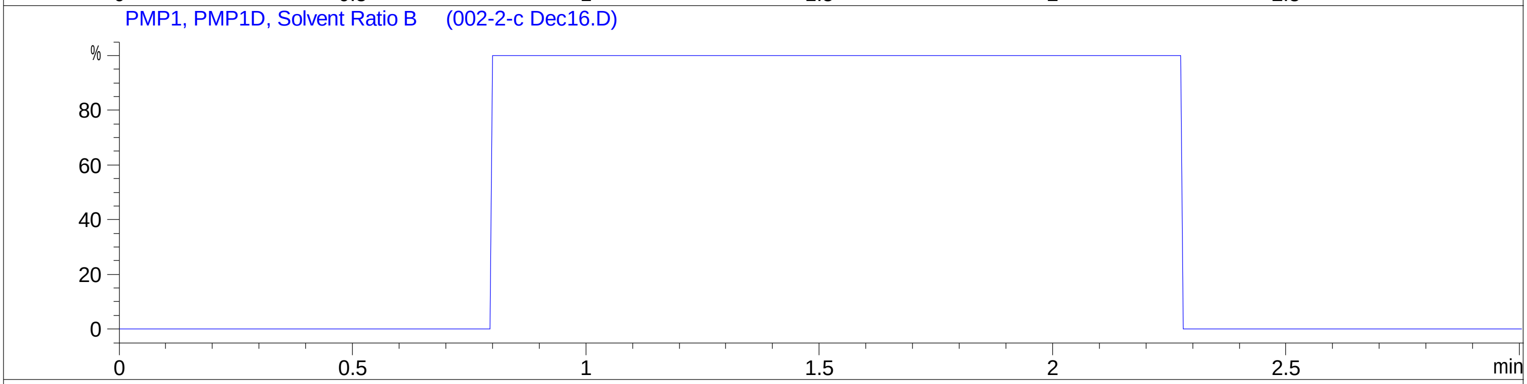

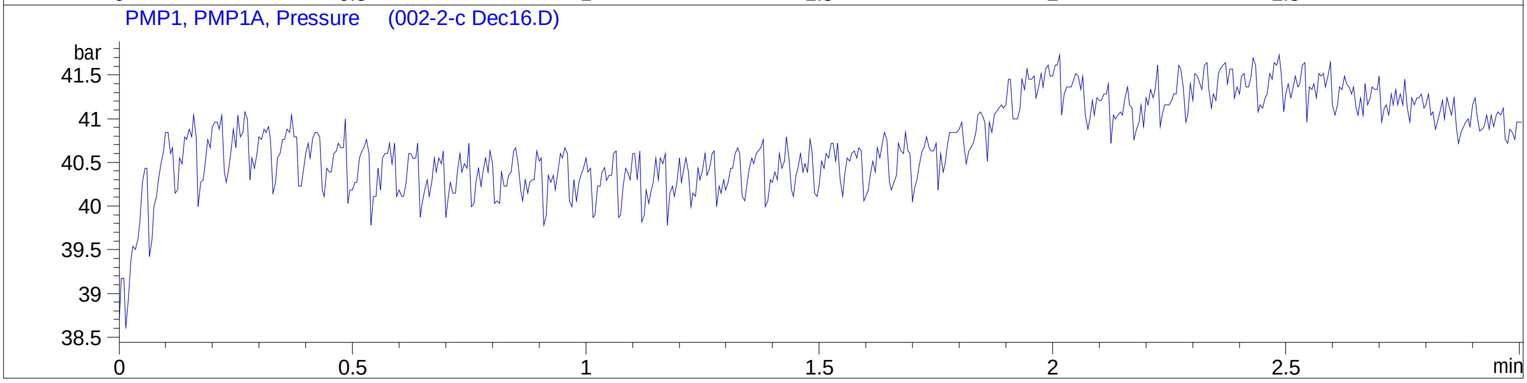

===

Area Percent Report

===

Sorted By : Signal
Multiplier : 1.0000
Dilution : 1.0000
Use Multiplier & Dilution Factor with ISTDs

Signal 1: MWD1 C, Sig=210,8 Ref=360,100

Peak #	RetTime [min]	Type	Width [min]	Area [mAU*s]	Height [mAU]	Area %
1	0.065	BV	0.0266	37.60262	21.10251	0.0649
2	0.182	VV	0.0603	1.19076e4	3134.03320	20.5663
3	0.245	VV	0.2081	4.41009e4	3173.05957	76.1688
4	2.164	VB	0.0722	1852.76257	398.96448	3.2000

Totals : 5.78989e4 6727.15976

===

*** End of Report ***
